# Hepatitis B Screening and Immunity Gaps in Cambodia: Should Vaccination Be Mandated for Healthcare Workers and Health Sciences Students?

**DOI:** 10.5334/aogh.5207

**Published:** 2026-05-11

**Authors:** Virak Sorn, Ian L. Rouse, Daravuthy On

**Affiliations:** 1 Faculty of Health Sciences and Biotechnology, University of Puthisastra, Phnom Penh, Cambodia; 2 President’s Office, University of Puthisastra, Phnom Penh, Cambodia; 3 University of Puthisastra Medical Clinic, University of Puthisastra, Phnom Penh, Cambodia

**Keywords:** hepatitis B virus (HBV), screening and vaccination, low- and middle-income countries (LMICs), healthcare workers, health sciences students, Cambodia

## Abstract

Hepatitis B virus (HBV) infection remains a major public health challenge globally, particularly in low- and middle-income countries (LMICs), where diagnosis, treatment, and vaccination coverage remain suboptimal. In Cambodia, despite more than two decades of inclusion of HBV vaccination in the national immunization program, protective immunity among healthcare workers and health sciences students is not routinely assessed. This policy-oriented perspective examines gaps in HBV immunity among health sciences students. Recent screening data from a Cambodian medical university (2025) reveal that more than half of students lack protective anti-HBs antibody levels, potentially exposing both healthcare providers and patients to preventable transmission risks. This article argues that Cambodia should strengthen nationwide HBV screening and consider mandatory vaccination and booster policies for high-risk groups—particularly healthcare workers and health sciences students. Such measures represent pragmatic institutional safeguards that are critical to reducing occupational transmission, preventing long-term liver disease, and aligning national efforts with the World Health Organization’s goal of eliminating HBV as a public health threat by 2030.

## 1. Introduction

Hepatitis B virus (HBV) infection remains a major global public health concern, affecting an estimated 254 million individuals worldwide and constituting a leading cause of chronic liver disease, cirrhosis, and hepatocellular carcinoma (HCC) [1, 2]. The global mortality burden attributable to viral hepatitis has increased substantially, rising from 1.1 million deaths in 2019 to 1.3 million in 2022, thereby ranking as the second leading cause of death from communicable diseases after COVID-19 [2]. In 2022 alone, HBV accounted for approximately 1.1 million deaths and 1.2 million new infections, with the majority of fatalities resulting from cirrhosis and HCC each year [2, 3].

Despite the availability of highly effective vaccines and antiviral therapies, HBV continues to pose a significant public health challenge, particularly in low- and middle-income countries (LMICs), where access to screening, diagnosis, and treatment remains limited [3, 4]. According to the World Health Organization (WHO) report published in 2024, a substantial gap persists across the HBV care cascade. Only 13% of individuals living with chronic hepatitis B (CHB) are aware of their infection status, and merely 3% are receiving treatment, despite an estimated 20% meeting eligibility criteria for therapy [2, 5]. This considerable treatment gap contributes to the ongoing high burden of HCC, which was estimated at 0.685 million cases in 2022 across East Asia, Southeast Asia, and North Africa [6].

In Cambodia, hepatitis B vaccination has been integrated into the national childhood immunization program for more than two decades, contributing to reduced transmission among younger cohorts. Nevertheless, emerging evidence suggests that childhood vaccination alone may not ensure sustained protective immunity into adulthood, particularly among individuals entering healthcare professions. Low awareness of hepatitis screening, inconsistent vaccination histories, and the absence of routine booster policies contribute to ongoing transmission risks within healthcare and training settings.

This policy perspective examines the current gaps in HBV screening and immunity in Cambodia, with a specific focus on healthcare workers (HCWs) and health sciences students. Drawing on recent institutional data, it explores whether targeted, mandatory HBV screening and vaccination policies should be considered as part of a broader hepatitis elimination strategy. The article argues that targeted, mandatory approaches for high-risk groups are both ethically justified and operationally feasible, and that they represent a critical step toward safeguarding patient safety, protecting the healthcare workforce, and advancing Cambodia’s commitment to the global hepatitis elimination agenda.

## 2. Hepatitis B Elimination Goals and the LMIC Challenge

The WHO has set an ambitious goal to eliminate viral hepatitis as a public health threat by 2030, aligned with the United Nations Sustainable Development Goals [2, 4]. However, recent modeling studies suggest that current trends are insufficient to meet these targets, with elimination potentially delayed until after 2050 if interventions are not intensified [7, 8]. Furthermore, only about 13% of individuals living with CHB are aware of their diagnosis, with very low rates of linkage to care—not only in LMICs but also in many high-income countries (HICs) [2, 8]. Patients with end-stage liver disease are more likely than those with other chronic conditions to be hospitalized, experience longer stays, and have higher readmission rates [9]. Notably, individuals with decompensated HBV infections often require frequent hospitalization, which increases the risk of virus transmission to healthcare providers if appropriate immunity and prevention strategies are not in place. HCWs face an elevated risk of HBV infection due to occupational exposure to blood and body fluids. Global estimates indicate wide variation in HBV prevalence among HCWs, ranging from less than 1% in Europe to over 8% in parts of Asia and sub-Saharan Africa—regions where vaccination coverage is often low [10]. These findings underscore the importance of occupational vaccination policies as a cornerstone of hepatitis elimination strategies.

## 3. The Cambodian Context: Gaps in Protection

Cambodia, situated in the WHO’s Western Pacific Region, has a high prevalence of HBV infection, affecting approximately 6.2% of adults. This region represents over 45% of the global HBV burden, with prevalence rates among member states varying significantly from 1% to 18.8% [11]. Cambodia has achieved substantial progress in integrating HBV vaccination into its national childhood immunization strategy over the past two decades. Nevertheless, emerging evidence indicates that protective immunity among adults—particularly those entering healthcare professions—remains suboptimal, highlighting persistent gaps in long-term HBV prevention and control.

A 2024 study among Cambodian HCWs reported HBsAg positivity of 4.9% with HBV vaccination coverage of only 59.3% [12]. Similar findings from Kampot and Kep provinces showed that approximately 40% of participants remained unvaccinated against HBV [13]. These data highlight ongoing risks of occupational and healthcare-associated transmission.

Additionally, health sciences students are required to engage in clinical practice in hospitals and clinics; therefore, pre-placement health checks for anti-HBs antibodies are necessary to ensure both safety and preparedness. This approach is now applied not only in Cambodia but also globally, especially in HICs.

In response to this gap, the University of Puthisastra implemented a comprehensive HBV screening initiative in 2025 for all first-year students in both bachelor’s and associate degree programs. The aim was to screen for HBsAg and anti-HBs with a titer prior to clinical placement. Among 666 screened students, only 32.9% demonstrated fully protective anti-HBs antibody levels (≥50 IU/L). While 11.0% had partial immunity (between <50 IU/L and ≥10 IU/L), 55.71% lacked protective immunity (<10 IU/L), which required a booster dose and a full three-dose vaccination series. “A higher threshold (≥50 IU/L) was used to reflect sustained protection in individuals at ongoing occupational risk.” Notably, three students (0.45%) tested positive for HBV infection. All students without protective antibodies or requiring a booster are now mandated by the university to receive free vaccination, diagnostic testing, and appropriate follow-up care to prevent further transmission. These findings provide rare, real-world evidence of substantial immunity gaps among future HCWs in Cambodia and raise concerns regarding patient safety, student health, infection prevention and control within training hospitals, and the long-term effectiveness of childhood vaccination.

## 4. Rethinking Vaccination Policy: Should it be Mandatory?

The institutional data on HBV screening in 2025 underscore an urgent need for comprehensive HBV screening campaigns and vaccination advocacy initiatives across Cambodia, as recent reports indicate persistently low HBV vaccination coverage—driven by lack of knowledge, attitudes, and awareness regarding HBV transmission, vaccination, diagnosis, and treatment [12, 13]. Although a nationwide childhood HBV vaccination program has been in place for several years, its long-term effectiveness appears suboptimal, warranting further assessment and booster doses to maintain protective antibody levels.

Mandatory HBV vaccination for HCWs and health sciences students is standard practice in many HICs and has been shown to substantially reduce occupational transmission rates [10]. In Cambodia, where healthcare-associated infections remain a concern, similar policies could yield significant public health benefits. Mandatory policies must be accompanied by free access to vaccination, confidentiality safeguards, and structured clinical follow-up.

From a policy perspective, mandatory HBV screening followed by vaccination or booster doses for non-immune individuals is a cost-effective preventive strategy. Prioritizing an anti-HBs antibody threshold of at least 50 IU/L may provide enhanced and sustained protection for those at high occupational risk. Universities and healthcare institutions represent practical entry points for implementation, supported by national guidelines and public financing mechanisms.

While universal adult screening may be challenging in LMIC settings, targeting high-risk groups—such as HCWs, health sciences students, and individuals with frequent healthcare exposure—represents a pragmatic and ethically justified approach to reduce transmission and strengthen health system resilience.

## 5. Implications for Practice and Policy

Strengthening HBV screening and vaccination policies in Cambodia would contribute to multiple health system goals: protecting the healthcare workforce, reducing transmission within healthcare settings, preventing progression to advanced liver disease, and lowering long-term treatment costs associated with cirrhosis and HCC.

Medical and health sciences institutions play a pivotal role in this effort. Embedding mandatory HBV screening and vaccination into pre-clinical requirements reinforces professional responsibility, patient safety, and infection prevention principles early in training. Such measures also align medical education with global best practices and occupational health standards.

To achieve global elimination targets, all adults should be screened at least once, with immediate treatment for those who test positive. This effort will be essential to scale up diagnosis and treatment and reduce the overall burden of HBV. This could be advantageous not only for Cambodia but also contribute to the WHO’s goal to eradicate HBV from all countries.

## 6. Limitations

This article presents a policy-oriented perspective informed by institutional screening data and therefore has several limitations. The screening results are derived from a single private medical university and may not be fully generalizable to all health sciences students or HCWs in Cambodia. Vaccination histories were not always verifiable, and long-term follow-up of antibody persistence after booster administration was not assessed. In addition, the study was not designed to evaluate clinical outcomes or cost-effectiveness of mandatory vaccination policies. Nevertheless, the findings provide important real-world evidence of immunity gaps in a high-risk population and highlight areas requiring further national-level research and surveillance.

## 7. Conclusions

Despite longstanding childhood immunization efforts, significant gaps in HBV immunity persist among HCWs and health sciences students in Cambodia. This perspective argues that nationwide HBV screening and mandatory vaccination or booster policies for high-risk groups should be urgently considered. Rather than framing such measures as sweeping national mandates, they should be viewed as pragmatic institutional safeguards that complement national hepatitis strategies and strengthen the future health workforce. Such measures would accelerate progress toward hepatitis elimination, enhance patient safety, and align Cambodia’s health system with global best practices in infection prevention and control. Targeted, mandatory HBV screening and vaccination for high-risk groups represent a feasible and evidence-informed step toward achieving hepatitis elimination goals in Cambodia.
